# Clinical efficacy of percutaneous nephrolithotomy versus retrograde intrarenal surgery for pediatric kidney urolithiasis: A PRISMA-compliant article

**DOI:** 10.1097/MD.0000000000008346

**Published:** 2017-10-27

**Authors:** Pei Lu, Rijin Song, Yuzhou Yu, Jie Yang, Kai Qi, Rongzhen Tao, Keliang Chen, Wei Zhang, Min Gu

**Affiliations:** a Department of Urology, First Affiliated Hospital with Nanjing Medical University; b Department of Urology, Nanjing Lishui People's Hospital, Nanjing, China.

**Keywords:** meta-analysis, pediatric, percutaneous nephrolithotomy, retrograde intrarenal surgery, urolithiasis

## Abstract

**Background:**

Percutaneous nephrolithotomy (PCNL) and retrograde intrarenal surgery (RIRS) are widely used for pediatric upper tract stones; however, comparisons of their clinical efficacies are needed.

**Methods:**

Literature searches for relevant articles were performed using PubMed, the Cochrane Central Register of Controlled Trials, Embase and the China CNKI database. Study quality was assessed by Jadad and Newcastle–Ottawa Scales. Standard mean differences (SMDs) or odds ratios (OR), and 95% confidential intervals (95% CIs) were pooled for meta-analysis. In addition, data was evaluated the quality of the body of evidence by means of grading of recommendations assessment, development, and evaluation (GRADE).

**Results:**

Data from 4 studies (231 PCNL, 212 RIRS cases) were analyzed. There was no significant difference in operation time (SMD: 1.39; 95% CIs: −0.049 to 2.82; *P* = .058), overall stone-free rate (OR: 3.72; 95% CIs: 0.55–25.22; *P* = .18), or complication rate (OR: 1.92; 95% CIs: 0.90–4.07; *P* = .091). PCNL cases had longer hospital stays (SMD: 1.22; 95% CIs: 0.95–1.50; *P* < .001), but showed a higher stone-free rate for stones greater than 20 mm (OR: 6.38; 95% CIs: 1.83–22.22; *P* = .004). For stones less than 20 mm, however, no significant difference between PCNL and RIRS was found (OR: 0.92; 95% CIs: 0.33–2.55; *P* = .87). The quality of evidence based on the GRADE system was low.

**Conclusion:**

Results of our systematic review and meta-analysis suggest that, for the treatment of larger kidney stones (>20 mm) in pediatric patients, PCNL is a better option due to its higher stone-free rate, although RIRS may be associated with shorter hospital stays. A large-scale clinical trial is necessary to validate our findings.

## Abstract 2

Supplemental Digital Content is available in the text

## 1 Introduction

Pediatric kidney stone disease is an important health problem worldwide, with increasing incidence and morbidity rates, especially in developing countries.^[1]^ Due to the advancement of surgical techniques and miniaturization of surgical instruments, management of upper urinary tract stones in children has dramatically changed in recent years.^[2,3]^ As a result of these improvements, pediatric kidney stones that were previously only treated by open surgery or extracorporeal shock wave lithotripsy (ESWL) can now be managed with various minimally invasive techniques, such as percutaneous nephrolithotomy (PCNL) and retrograde intrarenal surgery (RIRS).

The introduction of PCNL offered a novel approach to be considered for difficult cases instead of traditional open surgery. It has been reported that PCNL is more efficacious and less invasive, and is associated with less blood loss than open surgery, which was said to be performed more often in pediatric patients.^[4,5]^ For larger kidney stones (greater than 20 mm), PCNL has been recommended as the first-line treatment in adults.^[6]^ At the same time, the development of smaller endoscopes for stone disintegration has resulted in ureteroscopy being more frequently used to treat kidney stones in children. Even though ureteroscopic techniques are not recommended by current guidelines, RIRS, the representative endoscopic technique in the field, has been increasingly employed in various urology centers. In fact, RIRS may become a more attractive option than ESWL.^[7,8]^ However, consequences of the use of ureteral endoscopic methods in the pediatric population remain largely unknown, resulting in much debate about the clinical safety and efficacy of PCNL and RIRS in the treatment of pediatric kidney stones.

We present findings from a systematic review and meta-analysis of the clinical efficacy of PCNL and RIRS for kidney stones in children. To the best of our knowledge, there have been no such prior studies comparing PCNL and RIRS in pediatric kidney stone management.

## 2 Methods

### 2.1 Ethic statement

The study protocol was in accordance with the ethical standards of the Declarations of Helsinki and Istanbul, and the protocol of this study was approved by the local ethics committee of the First Affiliated Hospital with Nanjing Medical University.

### 2.2 Literature search

Two authors (PL and RS) performed a comprehensive literature search using PubMed, the Cochrane Central Register of Controlled Trials (CENTRAL), Embase, and China CNKI database (updated on April 1, 2016) to identify all potentially relevant studies. The following search items were used: (“pediatric” OR “child” OR “children” “pediatrics[mesh]”), AND (“percutaneous nephrolithotomy” OR “nephrolithotomy, percutaneous[mesh]”), AND (“retrograde intrarenal surgery”). Furthermore, the reference lists of all studies that were included in the meta-analysis and the abstracts of annual meetings of the American Society of Urology and European Association of Urology were reviewed.

### 2.3 Inclusion and exclusion criteria

The criteria used to select studies for meta-analysis were the inclusion of subjects within the appropriate age range (1–18 years); the use of a randomized control trial (RCT) or case-control study design; a focus on the safety and clinical efficacy of PCNL versus RIRS in pediatric kidney stone disease; the presence of at least 1 outcome variable of interest for our study. The exclusive criteria were listed as follows: case report, reviews, or letters to editors; articles written in languages other than English or Chinese; duplicate publications; studies lack of sufficient data. Two authors (PL and YY) used the above criteria to assess and select trials for the final analysis independently, with divergence of opinions settled by consensus. If such information was missing, we contacted the authors of the eligible studies. If we did not receive the requisite data, we excluded the study.

### 2.4 Data extraction and quality assessment

Relevant data from all eligible studies were extracted independently by 2 reviewers (PL and RS), and any discrepancies were resolved via consensus. The data collected included the first author, location of the study (country), publication year, study design, demographic data, mean operation time, mean stone-free rate, mean stone size, and mean hospital stay. For continuous results, the means (±SDs) were recorded for meta-analysis; whereas, for binary data, the number of subjects in each group was noted. To evaluate the quality of evidence, we performed the grading of recommendation assessment, development and evaluation (GRADE).^[9–11]^

The quality of all eligible studies was evaluated by 2 independent reviewers (PL and RS). For retrospective case-controlled studies, a checklist from the Newcastle–Ottawa Scale (NOS) was used; for RCTs, quality was assessed according to the Jadad Scale.

### 2.5 Statistical analysis

Pooled data were used to compare the safety and efficacy data (standard mean difference [SMD] or odds ratio [OR] with 95% confidence intervals [95% CIs]) for PCNL versus RIRS. *P* < .05 was considered statistically significant. Heterogeneity among trials was determined by *I*^*2*^, which is defined as 100% × (Q – df)/Q, where Q is Cochran heterogeneity statistic, and df is the degrees of freedom; a fixed-effect model set at low statistical inconsistency (*I*^*2*^* *< 25%) was used.^[12]^ Otherwise, we selected a random-effects model, which is better adapted for clinical and statistical variations. Sensitivity analysis was also performed to evaluate the stability of the meta-analysis. Briefly, a new analysis was done after omitting 1 study at a time to test study influence on the overall estimate. To explore the source of heterogeneity, subgroup analysis was performed, such as stone size and ethnicity. All the statistical analyses were performed using Stata Statistical Software: Release 12.0 (StataCorp LP, College Station, TX).

## 3 Results

### 3.1 Study characteristics

**Figure 1:**
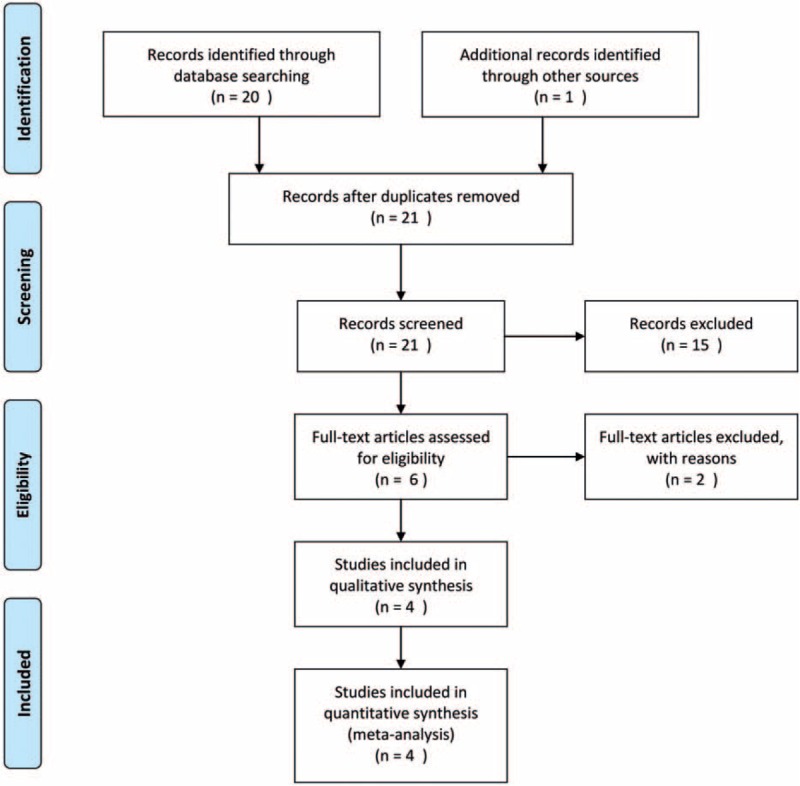
Flow diagram of the study selection process.

The basic characteristics of eligible studies are shown in Table 1. Four studies including 231 PCNL cases and 212 RIRS cases were selected for systematic review and meta-analysis (Fig. 1). There was no difference between PCNL and RIRS study populations in terms of age or sex ratio. All 4 studies were case-controlled studies and included 3 retrospective case-control studies^[13–15]^ and 1 RCT.^[16]^

**Table 1:**
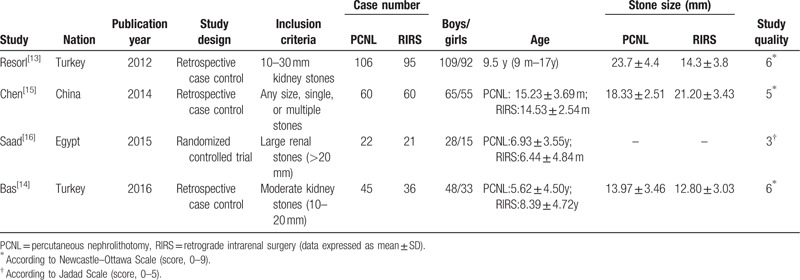
Characteristics of the included studies.

### 3.2 Meta-analysis results

**Figure 2:**
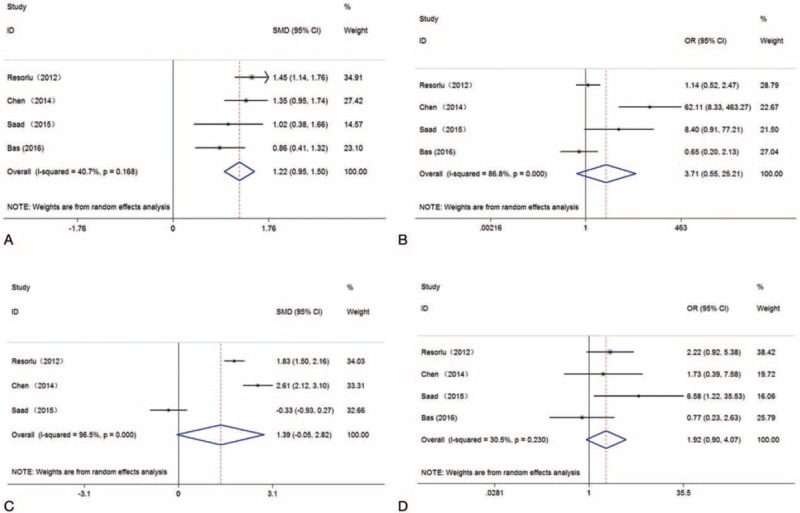
Forest plots for overall analyses: (A) hospital stay; (B) stone-free rate; (C) operation time; (D) complication rate.

As shown in Fig. 2, there was no significant difference between PCNL and RIRS groups for mean operation time (SMD: 1.39; 95% CIs: −0.049 to 2.82; *P *= .058), stone-free rate (OR: 3.72; 95% CIs: 0.55–25.22; *P *= .18), or complication rate (OR: 1.92; 95% CIs: 0.90–4.07; *P *= .091). The PCNL group had significantly longer hospital stays (SMD: 1.22; 95% CIs: 0.95–1.50; *P *< .001). With respect to these 3 case-control trials, no statistical significance was shown for stone-free rate (OR: 3.04; 95% CIs: 0.30–30.71; *P *= .35) or complication rate (OR: 1.60; 95% CIs: 0.85–3.01; *P *= .15) (Table 2). Similarly, we also found that the hospital stay in PCNL group was notably longer (SMD: 1.25; 95% CIs: 0.92–1.58; *P *< .001) and mean operation time (SMD: 2.20; 95% CIs: 1.44–2.96; *P *< .001) (see Supplementary Fig 1).

**Table 2:**
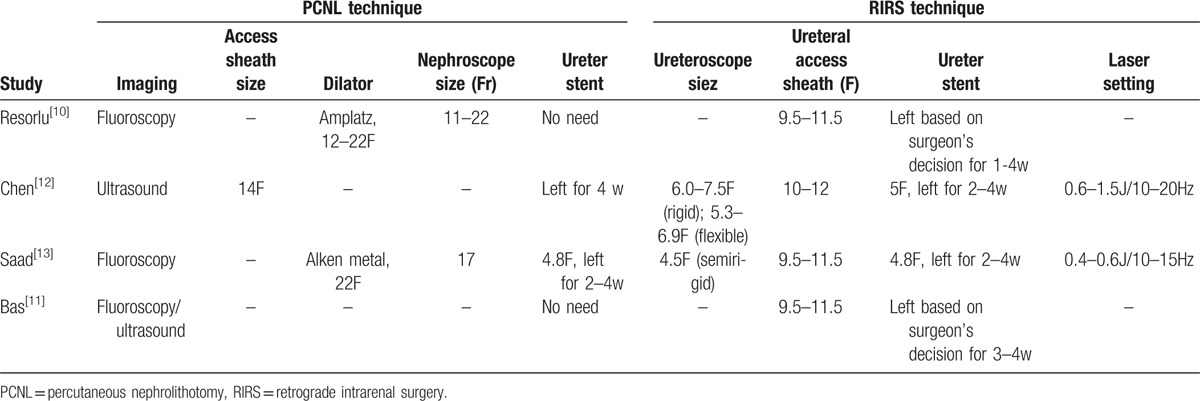
Overview of details for the percutaneous nephrolithotomy procedures and retrograde intrarenal surgeries reported in the included studies.

**Figure 3:**
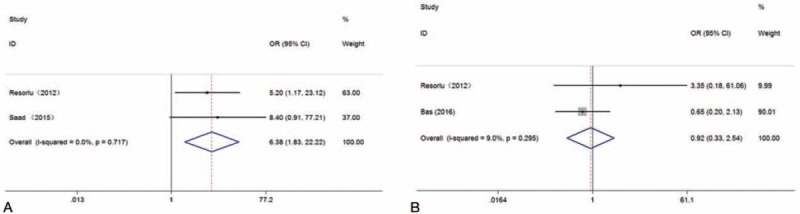
Forest plots for subgroup analyses for stone size: (A) stone size more than 20 mm; (B) stone size less than 20 mm.

For kidney stones greater than 20 mm, PCNL showed a statistically higher stone-free rate (OR: 6.38; 95% CIs: 1.83–22.22; *P *= .004); whereas, for kidney stones less than 20 mm, there was no significant difference between PCNL and RIRS (OR: 0.92; 95% CIs: 0.33–2.55; *P *= .87) (see Fig. 3).

As shown in Table 3, the total complication rates in the PCNL group among 4 studies ranged from 13% to 22%, while the rate ranged from 5% to 16% in the RIRS group, and no study reported the significant difference in the complication rate between 2 groups. Moreover, the most common complication among these studies was blood transfusion in the PCNL group and fever in the RIRS group, which should gain more attention for the postsurgery nursing and observation.

**Table 3:**
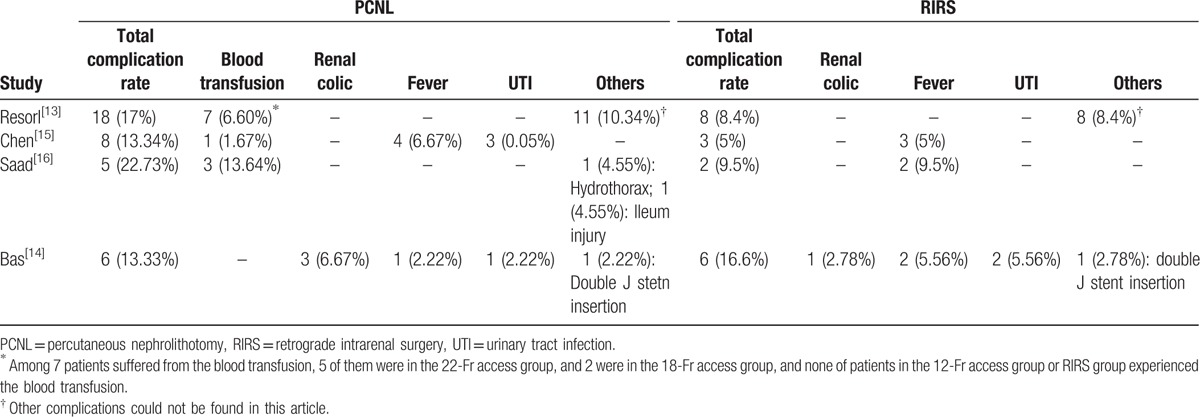
Postoperative complications for percutaneous nephrolithotomy and retrograde intrarenal surgery in the included studies.

### 3.3 Quality of evidence

Based on the GRADE summaries, which depends on the quality, consistency, directness and effect size, and the quality of the evidence regarding treatment effect, we deemed the quality of the evidence in various comparisons to be low or very low (Supplemental Table 1).

## 4 Discussion

The results of our study suggest that PCNL, although associated with a longer hospital stay, has a higher stone-free rate compared with RIRS when used to treat kidney stones greater than 20 mm in children. However, no difference was detected in terms of operation time, total stone-free rate, and complication rate.

Percutaneous surgery originated in the 1980s and has become the preferred surgical procedure for large kidney stones.^[17]^ In traditional PCNL for adult kidney stones, a 28F to 30F working sheath was placed to facilitate irrigation: this contributed to a high stone-free rate and a reduced surgical morbidity compared with traditional open surgery.^[18]^ Then, utilization of a working sheath with a smaller diameter attracted the attention of numerous urologists, and “mini”-percutaneous nephrolithotomy (mini-PCNL) was introduced.^[19,20]^ This procedure, which employs a 7F rigid cystoscope and 11F vascular access sheath, has been progressively applied in the management of adult and pediatric kidney stone diseases. In all 4 eligible trials in our analysis, the micro-perc was used to treat pediatric kidney stones. Moreover, with the advancement of flexible ureteroscopy and the introduction of endoscopic basket devices and flexible lithotrites, the technique of RIRS has become a novel weapon for pediatric urolithiasis.^[21]^ However, it should be noted that the small ureteric size in RIRS may not allow the passage of large access sheath and flexible ureteroscopy, which means an initial period of stenting may be needed during the RIRS surgery. And this shortage of RIRS has been reported in several studies.^[16]^ Both PCNL and RIRS have provided alternatives to traditional ESWL and open surgery in the management of pediatric kidney stones, and various studies have reported the successful treatment of larger kidney and upper tract stones by the PCNL procedure or RIRS.^[22–24]^

The key result of the present study is that PCNL showed a significantly higher stone-free rate in the treatment of pediatric kidney stones greater than 20 mm when compared with RIRS, a finding supported by a study by Woodside et al,^[17]^ suggesting the stone size to be an important factor for the stone-free rate of the PCNL and RIRS surgeries. However, the overall stone-free rates for PCNL and RIRS cases were not significantly different. After the size decrease of the working sheath in PCNL, increasing numbers of urologists showed interest in the procedure. Desai et al^[25]^ reported their experience using PCNL for pediatric renal calculi in 56 patients and achieved a nearly 90% stone clearance rate with PCNL monotherapy. Later, Yan et al^[26]^ showed a complete clearance rate of 85.2% for renal calculi in preschool age children using mini-PCNL monotherapy. Likely, the stone-free rate declined dramatically in children with more than 2 stones or increased stone size (>20 mm).^[27]^ Moreover, 1 single-center study reported a 94.0% stone-free rate at hospital discharge after mini-PCNL for kidney and upper tract stones in pediatric patients less than 3 years of age.^[24]^ In our meta-analysis, the overall stone-free rate for the PCNL group was 88.84%, whereas the stone-free rates for smaller (<20 mm) and larger stones (>20 mm) were 84.5% and 86.1%, respectively, similar to previously reported studies (75%–100%). Not limited to the stone size of patients, various factors, such as the number of stones, the shape of stones, have been reported to have potential impacts on the procedure of RIRS and PCNL surgeries, consequently affecting the stone-free rate. The study conducted by Resorlu et al^[13]^ showed that 37% of stones in PCNL group were multicalyces compared with the 9.5% in the RIRS group. Similarly, it was reported that in the PCNL group, 24% staghorn stones were included for surgery, whereas the rate was 14% in the RIRS group.^[16]^ However, we could not perform the subgroup analysis based on the influence of these factors on the stone-free rate due to the limited data. Recently, a study comparing the mini-PCNL surgery in the preschool children and infants reported that compared with ESWL, the mini-PCNL with a 77% primary stone-free rate and 92% final stone-free rate could be considered an effective minimally invasive procedure for pediatric kidney stones.^[27]^ Therefore, as mini-PCNL is characterized as a minimally invasive procedure, it remains the first-line procedure for pediatric kidney stones, especially for stones larger than 20 mm because of its significantly higher stone-free rate.

Our meta-analysis did not reveal a difference between the operation times for PCNL and RIRS procedures. Operation time is associated with the urologist's surgical technique, the instruments used, and the stone size. Bryniarski et al^[28]^ reported a 20-minute longer mean operation time for standard PCNL, and suggested that the increased procedure time was due to the necessary use of telescopic dilators and ultrasonic lithotripsy. A similar result was also found by Resorlu et al,^[13]^ believed to result from the time needed to obtain percutaneous renal access under fluoroscopic guidance. Interestingly, the opposite results were obtained in a RCT by Karim:^[16]^ a longer procedure time for RIRS was attributed to a larger average stone size and mandatory double-J stent placement. More importantly, the pooled results of operation time between 2 surgeries should be interpreted by caution because the operation time in RIRS surgery should be more than that in the PCNL group when the stone sizes of 2 groups are comparable. Thus, we reasonably believed that the operation time in RIRS surgery should be less than that in the PCNL group when the stone sizes of 2 groups are comparable.

Our study found longer hospital stays in the PCNL group than in the RIRS group, possibly related to a higher complication rate after PCNL—as was reported from a meta-analysis by De et al^[29]^—even though no significant difference between the complication rates for the procedures was found in ours. The general complication rates for the PCNL and RIRS groups were 16.31% and 8.96%, respectively. All complications were described as minor (Clavien I–II). The most frequent complications in the PCNL group were the need for blood transfusion and renal colic; whereas, fever and urinary tract infections were most common in the RIRS group. Importantly, there were 10 pediatric patients in the PCNL group with intraoperative bleeding requiring blood transfusion, but none in the RIRS group. In addition, chest complications are known to sometimes occur after supracostal percutaneous access attempts.^[30]^ Our systematic review included 1 patient who developed hydrothorax after supercostal puncture that resolved without chest tube placement, whereas no complication related to the application of ureteric access sheath was reported in the RIRS group. Therefore, certain precautions should be recommended with PCNL to reduce the chest complication rate such as use of an Amplatz sheath or intraoperative chest fluoroscopy.^[30]^

Some factors may have affected our results and serve as limitations of this study. First, there were a small number of included trials of relatively poor quality. The heterogeneity among these studies was relatively high, which may be explained by the study designs, surgical techniques, clinical practices, and outcome definitions. Then, due to the deficiency of the eligible data among these trials, we could not perform the subgroup analysis of these main characters, such as operation time, complication rate and the influence of the ethnicity and surgical techniques on these surgeries. Moreover, as reported previously, the number of stones, the shape of stones, and the number of calyces may have significant impact on the stone-free rate. Limited to extractable data, a large-scale and well-designed clinical study should be conducted to further explore the influence of these factors on the RIRS and PCNL surgeries.

In conclusion, results from our systematic review and meta-analysis suggest that the difference in the kidney stones could have the significant impact on the stone-free rate. With regard to this, we suggest the stone-free rate of PCNL therapy is better for pediatric patients. Moreover, PCNL monotherapy is a better option for the treatment of larger kidney stones (>20 mm) in pediatric patients due to its higher stone-free rate, whereas RIRS had the advantage of a shorter hospital stay. A large-scale, well-designed clinical trial should be carried out to confirm these findings.

## Supplementary Material

Supplemental Digital Content
