# Supplementary material for: Clinical efficacy of percutaneous nephrolithotomy versus retrograde intrarenal surgery for pediatric kidney urolithiasis: A PRISMA-compliant article
Source: Medicine (Baltimore). 2017 Oct 27;96(43):e8346. doi: 10.1097/MD.0000000000008346 (PMC5671844; doi:10.1097/MD.0000000000008346)
Supplement: Supplemental Digital Content [file medi-96-e8346-s001.docx]

Supplemental Fig 1. Forest plots for meta-analysis in case-control trials: (A) hospital stay; (B) stone-free rate; (C) operation time; (D) complication rate.

**
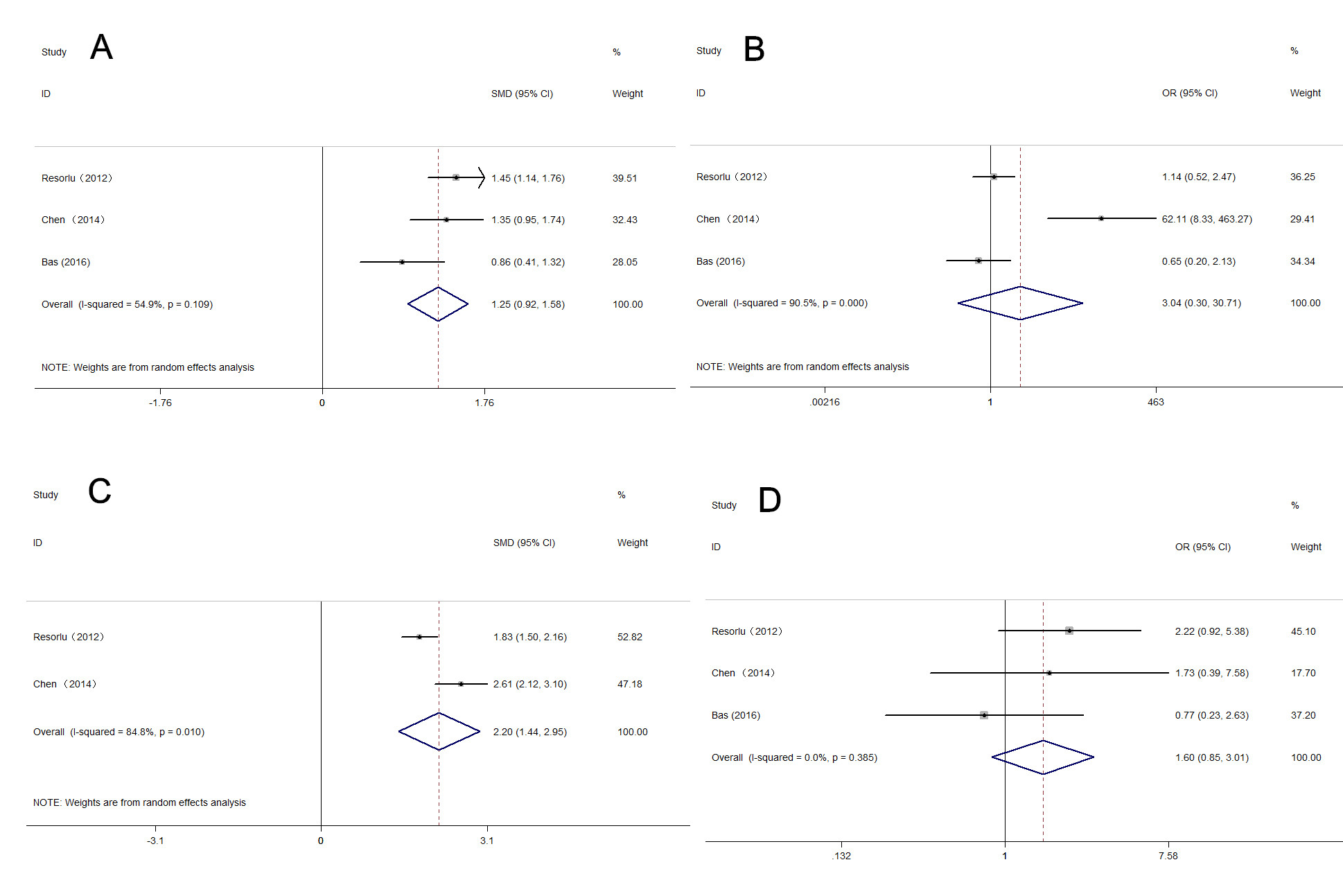
**

**Supplemental Table 1. Results of the GRADE recommendation to evaluate the overall quality of evidence in our meta-analysis.**

| **Comparisons** | **Type of evidence** | **Quality** | **Consistency** | **Directness** | **Effect size** | **Total scores** |
| --- | --- | --- | --- | --- | --- | --- |
| Complication rates | 2 | -1(with no blinding allocation process) | 0 | 0 | 0 (OR=1.92) | 1 (very low) |
| Hospital stay | 2 | -1(with no blinding allocation process) | +1 (adjustment for confounders would have increased the effect) | 0 | 0 (OR=1.22) | 2 (low) |
| Operation time | 2 | -1(with no blinding allocation process) | 0 | 0 | 0 (OR=1.39) | 1 (very low) |
| Stone-free rate | 2 | -1(with no blinding allocation process) | 0 | 0 | +1 (OR=3.71) | 2 (low) |
